# Sero‐Prevalence of Foot‐and‐Mouth Disease in Cattle in Selected Districts of Jimma Zone, South‐Western Ethiopia

**DOI:** 10.1002/vms3.70239

**Published:** 2025-02-22

**Authors:** Geremew Batu, Zelalem Abera, Moti Wakgari, Eshetu Gazagn

**Affiliations:** ^1^ West Wollega Livestock and Fisheries Development and Resource office, Gimbi Oromiya Regional Ethiopia; ^2^ Department of Veterinary Clinical Science and Laboratory Technology School of Veterinary Medicine Wollega University Nekemte Ethiopia; ^3^ Bedele Veterinary Regional Laboratory Bedele Ethiopia

**Keywords:** Cattle, ELISA, Ethiopia, FMD, Jimma, Sero‐prevalence

## Abstract

**Background:**

Foot‐and‐mouth disease (FMD) is highly contagious and results in a high economic loss in the world.

**Methods:**

A cross‐sectional study was conducted from December 2021 to June 2022 in three selected districts to determine the sero‐prevalence of FMD. A total of 384 cattle sera samples were collected and tested for antibodies against FMD virus using FMD NSP c‐ELISA.

**Results:**

The overall sero‐prevalence of 25.3% was determined. Sero‐prevalence of 31.0%, 32.37% and 4.5% was seen in the districts. Higher sero‐prevalence was observed in Limmu Seka, and the disease was statistically significant in both Limmu Kosa and Limmu Seka districts (*p* < 0.05). Higher sero‐prevalence was seen at Ambabesa Sadeka Peasant Association (42.86%). However, except Ambabesa Sadeka peasant association (PA), there was no statistically significant association (*p* > 0.05) of FMD occurrence among the nine PAs. Higher sero‐prevalence was recorded in females (33.65%), old age (39.1%), poor body condition (43.0%) and herd size (43.1%). Therefore, sex, age, body condition of the animals and herd size showed significant association (*p* < 0.05) in the occurrence of FMD in the study areas. Purchased animals were highly infected (56.4%), and there was also a statistically significant association in the origin of the animals (*p* < 0.05).

**Conclusion:**

Overall, FMD is an economically important disease in the study areas.

**Recommendation:**

Further studies are warranted to characterize FMD virus serotypes in the areas and investigation in wildlife and small ruminants is needed to determine their roles in FMD virus maintenance and transmission.

## Graphical abstract

Foot‐and‐mouth disease (FMD) is highly contagious and results in a high economic loss in the world. A cross‐sectional study was conducted from December 2021 to June 2022 in three selected districts of the Jimma zone to determine the sero‐prevalence of FMD. A total of 384 cattle sera samples were collected from randomly selected cattle of the study areas and examined for the presence of FMD virus using FMD NSP c‐ELISA. SPSS version 20 was used for data analysis. Descriptive statics techniques were applied. Out of the total serum samples collected, 97 were found positive for the disease with an overall sero‐prevalence of 25.3%. Higher sero‐prevalence was observed in Limmu Seka, and the disease was statistically significant in Limmu Kosa and Limmu Seka districts. Overall, FMD is an economically important disease in the study areas which needs further investigation to understand its transmission methods.

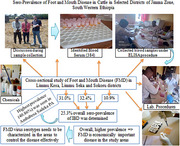

## 1 Introduction

The contribution of livestock to the national economy, particularly with regard to foreign currency earnings, is through the exportation of live animals, meat, skin, and hides (Ayele et al. 2003). However, the development of this sector is hampered by various constraints. The most important constraints are widespread endemic diseases including viral, bacterial and parasitic infestation (Abdela 2017). Among the health constraints, foot‐and‐mouth disease (FMD) is one of the trans‐boundary animal diseases that cause significant loss in many species of livestock and livestock products (OIE 2009). FMD is a highly contagious viral disease that is transmitted from open‐hoofed animals and is one of the leading causes of economic loss and loss in cattle (Knight‐Jones and Rushton 2013). Foot‐and‐mouth disease virus (FMDv) O, A, C, South African type (SAT_1_, SAT_2_, SAT_3_) and Asia_1_ allegedly have seven serotypes known to cause disease (Knowles et al. 2016, Tekleghiorghis et al. 2016). Among these seven serotypes, serotypes O and A are the most common in Africa (Rweyemamu et al. 2008, Jemberu et al. 2016). The incidence of FMD due to serotype C seems to have decreased recently. Serotype C was last reported in Kenya in 2004 and in Ethiopia in 2008 (Roeder and Knowles 2008, Rufael et al. 2008).

Published articles on FMD show that types A and O are the major serotypes responsible for significant economic losses in the country (Gelaye et al. 2009). All seven serotypes cause clinically indistinguishable disease, but due to antigen diversity, immunity to one serotype does not provide protection against another (Bari et al. 2014). It is a contagious animal disease that affects livestock and wild ungulates and is susceptible to cattle, pigs, sheep, goats and buffalo (Paton, Gubbins, and King 2018). The disease is characterized by fever, loss of appetite, weight loss and blisters on the mucous membranes, especially the mouth, breasts and nipples (Admassu et al. 2015). FMDv is the pathogen of FMD and belongs to the genus *Aphthovirus* and the family *Picornavirus* (Knowles and Samuel 2003, Grubman and Baxt 2004). Based on sequence analysis of capsid proteins, serotypes are also assigned topotypes, indicating geographical, antigenic and genetic relationships between serotypes (Soltan et al. 2017). FMD is unique to Ethiopia, a member of the World Organization for Animal Health (OIE), and five of the seven (O, A, C, SAT_1_ and SAT_2_) FMD virus serotypes have been detected in several national research publications (Abdela 2017, Ayelet et al. 2013, Legesse et al. 2013).

The disease spreads rapidly by movement of infected animals or mechanically via fomites such as clothing, shoes, vehicles and veterinary instruments (Knight‐Jones and Rushton 2013). The reasons for its rapid spread to a completely sensitive population are the high infectivity of the virus, the production of high titers in respiratory secretions and the large amount of viral droplets and aerosols shed from infected animals. It is in the stability of the virus that it occurs. Such droplets have a fast replication cycle with very high virus yields and a short incubation time of the virus (Rweyemamu et al. 2008). FMD is the most important endemic disease in Ethiopia and has significant socio‐economic implications as a result of reduced production, newborn deaths, huge costs of veterinary services and restrictions on the movement of animals and meat between local and national regions (Knight‐Jones and Rushton 2013).

FMD, one of the endemic diseases of Ethiopia, recurs annually and causes several outbreaks (Ayelet et al. 2012). Serological studies in different parts of the country have reported sero‐prevalence of 5% to 27% at the animal level and up to 60% at the herd level (Rufael et al. 2008, Megersa et al. 2009, Bayissa et al. 2011). In Ethiopia, there has been recent interest in controlling FMD to promote the export of live animals and meat, as it is considered a major obstacle to the international trade of livestock and their products (Ministry of Agriculture and Rural Development (MOARD) 2006). However, controlling FMD in developing countries, especially in Ethiopia, is not an easy task for several reasons. These include the widespread distribution of multiple serotypes and subtypes, the presence of various hosts of the virus throughout the country, uncontrolled livestock movement and lack of sufficient amount of effective and affordable FMD vaccine in the country. In addition, FAO and OIE argue that the management of FMD in endemic countries such as Ethiopia must be carried out with a long‐term, progressive approach to risk reduction (Rweyemamu et al. 2008, Paton, Sumption, and Charleston 2009).

Ethiopia possesses a huge number of livestock populations in Africa, and the livestock sector covers 16.5% of the national gross domestic product (GDP) and 35.5% of the agricultural GDP (Livestock 2017). FMD causes several outbreaks every year and its impact has been increasing from time to time. In 2005–2006, Ethiopia lost about 14 million USD as a consequence of the Egyptian trade ban. Also, a trade ban in 2011 estimated the loss to be 3,322,269 USD as a result of bull rejection from the market. Economic losses recorded in terms of losses arising from milk loss, mortality and draft power were estimated to be 76 USD per affected herd, 9.8 USD per head in crop‐livestock mixed system, and 174 USD per affected herd and 5.3 USD per head in the pastoral system (Yalew 2019).

Disease endurance and constantly changing state could be caused by a lack of epidemiological knowledge about the illness and insufficient control methods in place. Therefore, it is necessary to clarify several epidemiological factors before initiating and implementing effective control and prevention measures. These factors typically include evaluating the prevalence of FMD, identifying some risk factors and understanding the disease's geographic distribution through efficient and frequent surveillance. Even though the epidemiology of the disease has been studied extensively, there is still a dearth of current, comprehensive data on sero‐prevalence and related risk factors. Furthermore, no comprehensive data about the disease prevalence in the Jimma zone exist. Consequently, the current study's goals were to ascertain the sero‐prevalence of FMD in cattle from several districts of the Jimma zone.

## 2 Materials and Methods

### 2.1 Description of the Study Area

Jimma is one of the Oromia National Regional State's 21 zones, where the study was carried out. The zone is 347 km away from Addis Ababa and consists of 21 districts. Astronomically, it is located between 7°40''N latitudes and 36°50''E longitudes and is an elevation of 1780 m. It is located in the south‐western part of Ethiopia, bordered on the south by the Southern Nations, Nationalities and Peoples region, on the northwest by the Illubabor zone, on the north by the East Wollega zone and on the northeast by the West Shewa zone; part of the boundary with the east Shewa zone is defined by the Gibe River. The mean annual temperature is fairly high. Generally, the mean annual temperature and rain fall of the zone varies from 24°C to over 27°C and 1200–2800 mm, respectively (World Weather Information Service 2019). The livestock population of the Jimma zone is 2,560,207 cattle, 859,914 sheep, 570,387 goats, 281,113 equines and 2,235,702 poultry (CSA 2012).

The study was carried out in the purposively chosen Jimma zone districts (Limmu Kosa, Limmu Seka and Sokoru) based on the recurring animal disease issues reported by these districts to the zone authorities. As a result, a survey was suggested in conjunction with Bedelle Regional Laboratory to determine the issue in the area.

The Limmu Kosa district is located in the Jimma zone of the Oromia region of Western Ethiopia, 439 km west of Addis Ababa, at latitude 08°30`617''N and longitude 036°94`117''E. The altitude ranges from 1200 to 3020 m above sea level. It is bordered on the south by Kersa, on the southwest by Mana, on the west by Gomma, on the northwest by the Didessa River, which separates it from the Illubabor zone, on the north by Limmu Seka, on the northeast by the Gibe River, which separates it from the West Shewa zone and the Southern Nations, Nationalities and Peoples Region, on the east by Sokoru, and on the southeast by Tiro Afeta. The district is characterized by a humid tropical climate with annual rainfall of 1600 mm and an average temperature of 17.5°C (Livestock 2017). The area is home to 248, 299 cattle, 197,254 sheep and 78,714 goats (LKDAO 2022). There are 44 peasant associations (PAs) in the area.

Limmu Seka district is found in the Jimma zone of Oromia state, Western Ethiopia, which is located 450 km west of Addis Ababa, at 09°29`N latitude and 037°26` E longitudes with an elevation of the district ranging from 1400 to 2200 m above sea level. Limmu Seka was bordered on the southwest by the Didessa River, which separates it from the Illubabor zone, on the northwest by the East Wollega zone, on the northeast by the Gibe River which separates it from the West Shewa zone, and on the southeast by Limmu Kosa. The average temperature varies from a minimum of 15°C to a maximum of 31°C. There are two distinct seasons in Limu Seka: the rainy season (from late March to October) and the dry season (November to early March). The rainfall is often more than 1,800 mm per annum (Livestock 2017). The district has a cattle population of 319,659, 177,902 sheep and 117,810 goats. The district has 42 PAs (LSDAO 2022).

**FIGURE 1:**
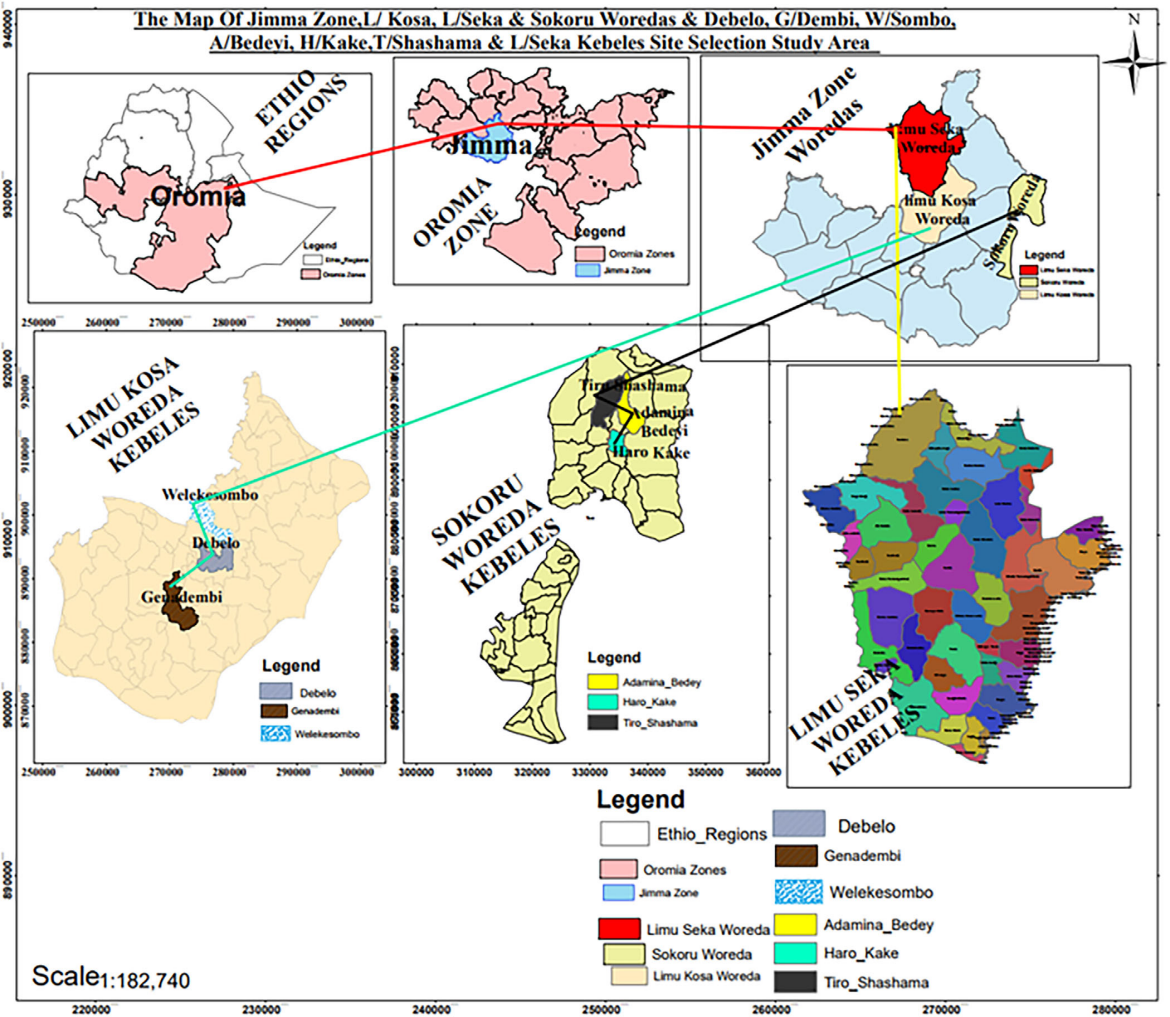
Map of the study area (projected using ArcGIS software).

Sokoru district is found in the Jimma zone of Oromia state, Western Ethiopia, which is located 263 km west of Addis Ababa, at 7°92`N latitude and 37°42`E longitudes with an altitude ranging from 900 to 2300 m above sea level. Sokoru is bordered on the south by Omo Nada, on the west by Tiro Afeta and the north and east by the Southern Nations, Nationalities and Peoples Region; the Gibe River defines the northern boundary. The maximum and minimum temperature of the district was 28.3°C and 12.1°C, respectively, while the average annual rainfall was 1,458 mm (Livestock 2017). The area has 234,568 cattle, 38,468 sheep and 48,654 goats (SDAO 2022). There are 41 PAs in the district (Figure 1).

### 2.2 Target and Study Population

The target population in this study were all local cattle greater than six months old from selected PAs of Limmu Kosa, Limmu Seka and Sokoru districts of the Jimma zone. Based on physical examination, sampled animals did not show any suggestive clinical signs of FMD such as vesicular eruptions in the mucosa of the mouth, salivation, fever or loss of appetite. According to Pace and Wakeman (2003), the age groups of cattle were categorized as (≤3.5 years) young, (3.5–5.5 years) adult and (>5.5 years) old. In addition, herd size was categorized as small (<40 cattle), medium (40–75 cattle) and large (>75 cattle) (Asresie, Zemedu, and Adigrat 2015). These study populations were reared in selected districts of the zone with the farming system of crop–livestock mixed and these animals are usually kept mixed with other animal species.

### 2.3 Study Design

A cross‐sectional study was conducted from December 2021 to July 2022. Variables included in the survey were study area, age (young, adult, old), animal origin (born, purchased), sex, herd size (small, medium, large), animal management, new animal introduction, herd composition, herd contact and herd contact area were recorded, and body condition score (BCS) was made based on Nicholson and Butterworth (1986) and animals were further categorized into three categories, with BCS 1, 2 and 3 as poor, BCS 4 and 5 as medium and BCS 6–9 as good, were emphasized as risk factors.

### 2.4 Sample Size Determination and Sampling Technique

The sample size was decided based on the formula described by Thrusfield (2018) with a 95% confidence interval at 5% desired absolute precision, and it is achieved by assuming the expected prevalence of 50%.

$$n=\frac{1.96^{2*}\mathrm{Pexp}\left(1-\mathrm{Pexp}\right)}{d^{2}},$$

where *n* is the sample size of the study population; *d* is the absolute desired precision of 5% and *p* is the expected prevalence of 50%.

$$n=\frac{1.96^{2*}0.5\left(1-0.5\right)}{0.05^{2}}=384.$$

Therefore, a sample size of 384 was considered for this study. First, districts were purposively selected based on cattle disease outbreak reports and the problem of livestock in the area and access to road facilities and individual animals and herds were selected by using a simple random sampling technique. Limmu Kosa, Limmu Seka and Sokoru districts have 44, 42 and 41 PAs, respectively. Based on the cattle population and the number of PA lists from each district, three PAs from Limmu Kosa district, three PAs from Limmu Seka and three PAs from Sokoru districts were randomly selected by the lottery method. In the second stage, the household of each PA was randomly selected. Finally, except for calves below 6 months, all cattle were randomly sampled (Table 1).

**TABLE 1:** Population of cattle and number of households per PA in the selected PA.

District	Peasant association (PA)	Number of households/PA	Number of cattle/PA	Number of selected Households	Number of samples taken per PA
Limmu Kosa	Waleke Sombo	652	5454	13	44
	Debelo	594	4328	9	40
	Gena Dembi	708	3668	7	42
Limmu Seka	Ambabesa Sadeka	680	6728	15	49
	Dora	517	4809	11	43
	Dame	449	3586	10	47
Sokoru	Tiro Shashema	565	4584	12	37
	Haro Kake	532	3298	11	40
	Adami Badeyi	687	4199	12	42
Total				100	384

### 2.5 Sample Collection and Transportation

About 10 millilitres of blood samples was collected from the jugular vein of each cattle using sterile vacutainer tubes and needles by following an aseptic procedure after the cattle was restrained by the owner, and each sample was properly labelled (including all necessary information such as owner name, species of animal, sex, age, breed, body condition, origin and herd size). The samples were kept protected from sun light in a slanting position overnight at room temperature to drain sera samples. The serum was separated manually and transferred to a sterile cryovials tube, labelled and transported to Bedelle Regional Laboratory Center under a cold chain for laboratory analysis. The sera were stored at −20°C until the test was performed. Competitive ELISA was used to detect FMD virus antibodies using a commercially prepared FMD NSP c‐ELISA test kit. Individual‐level related risk factors: sex, age, body condition and source of animals were collected using serum collection format.

### 2.6 Serological Test and Laboratory Analysis

Sera collected from cattle were tested by FMD virus 3ABC‐Ab ELISA (ID Screen FMD NSP Competition, 34790 Grabels, France) for the detection of antibody to 3ABC poly protein, which is a useful indicator of FMD virus infection. Antibody to 3ABC (nonstructural protein) is found only in virus‐infected animals but not in vaccinated animals. Therefore, the test was used to detect the presence of antibodies in the collected serum following the kit manufacturers’ recommended protocol. All the reagents, buffers, microplates and reactive and non‐reactive control sera were supplied by the manufacturer and the test was performed as per their guideline. First, all reagents were kept at room temperature and homogenized by vortexing. The test was carried out in 96‐well microplates. Briefly, 50 µL of the dilution buffer 18 was added to each well. 30 µL of the positive control were added to wells A1 and B1, and the same volume of negative control was also added to wells C1 and D1, while the rest wells were filled with 30 µL of test sera.

Then, the micro titer plate was covered and incubated for 2 hours ±10 min at 37°C (±3°C). After incubation, the sera were discarded from the plates, and each well was washed five times approximately by 300 µL of washing solution. After washing, 100 µL of conjugate 1× was dispensed into each well, and the micro titer plate was covered and incubated for 30 ± 3 min at 21°C (±5°C). After incubation, the sera were again discarded from the plates, and each well was washed five times approximately by 300 µL of washing solution. Then, 100 µL of the substrate solution (TMB) was dispensed into each test well and again incubated for 15 minutes at 21°C (±5°C) in the dark place. To stop colour reaction, 100 µL of the stop solution was dispensed into each well.

Finally, the optical density (OD) readings were recorded using a spectrophotometer at a wavelength of 450 nm. To check the validity of the FMD NSP Competitive ELISA result, a validity test was done. In valid FMD NSP c‐ELISA result, the mean OD value of the negative control serum was greater than 0.7 (OD_NC_ > 0.700), and the mean value of the positive control optical density (OD_PC_) was less than 30% of the OD_NC_ (OD_PC_/OD_NC_<0.3). For the interpretation of the result, the result of the test sera was expressed as an index, derived by dividing the absorbance value of the serum tested with reference to the OD of reference negative controls. For each sample, the competition percentage (S/N %) was calculated. The formula used to calculate present sero‐reactivity is

$$S/N\%=\frac{OD_{\mathrm{sample}}}{OD_{\mathrm{NC}}}\times 100.$$

Sample presenting S/N%: less than or equal to 50% are considered positive and greater than 50% are considered negative.

### 2.7 Data Management and Analysis

All data obtained from the field were recorded in the record sheet format and later entered into a Microsoft Excel worksheet, and descriptive and analytical statistics were carried out using SPSS version 20. Descriptive statistics was used to determine the frequency of proportion (prevalence). The total prevalence was calculated by dividing the number of FMD NSP c‐ELISA–positive animals by the total number of animals tested. The occurrence of association between the dependent variable (sero‐positivity) and independent variables was analysed by logistic regression analysis, and odds ratio (OR) was employed to assess the level effects of risk factors on FMD. In the analysis, the confidence level was held at 95 %, and *p* < 0.05 was set for significance.

## 3 Result

### 3.1 Overall Sero‐Prevalence of FMD in Cattle

A study on sero‐prevalence was conducted to determine the risk variables associated with the incidence of FMD within the study areas. Sex, age, body condition, herd size and animal origin were all included in the study as significant risk factors that contribute to FMD infection in the study region. A sero‐prevalence of 25.3% was observed in the research areas out of the 384 cattle that were investigated, with 97 cattle being determined to be sero‐positive overall.

### 3.2 District and PA Level of Sero‐Prevalence

#### 3.2.1 District Level of Sero‐Prevalence

To ascertain the sero‐prevalence of FMD in cattle, the study was carried out in three districts of the Jimma zone: Limmu Kosa, Limmu Seka and Sokoru. The respective sero‐prevalences of the disease in cattle were recorded as 31.0%, 32.4% and 10.9% in Limmu Kosa, Limmu Seka and Sokoru districts, respectively. Animals from Limmu Kosa and Limmu Seka districts had a nearly four‐fold higher likelihood of having FMD (OR = 3.7; 95% CI: 1.8–7.3; *p* = 0.000 in Limmu Kosa and OR = 3.9; 95% CI: 1.9–7.7; *p* = 0.000 in Limmu Seka) than those from Sokoru district (Table 2). As a result, the disease was statistically significant (*p* < 0.05) among those animals.

**TABLE 2:** Sero‐prevalence of FMD at the district level.

Study districts	No. of examined	No. of positive	Prevalence (%)	*P* value	OR	95% CI	
						Lower	Upper
Limmu Kosa	126	39	31	0.000	3.7	1.8	7.3
Limmu Seka	139	45	32.4	0.000	3.9	1.9	7.7
Sokoru	119	13	10.9	—	—	—	—
Total	384	97	25.3	—	—	—	—

#### 3.2.2 PA Level of Sero‐Prevalence

Sero‐prevalence of FMD at the PA level was studied in which different sero‐prevalence rates of the disease were observed among nine PAs of the three districts with 40.9%, 22.5%, 28.6%, 42.9%, 23.3%, 29.8%, 10.8%, 7.5% and 14.3% from Walake Sombo, Debelo, Gena Dembi, Ambabesa Sadeka, Dame, Dora, Tiro Shashema, Haro Kake and Adami Badeyi PAs of Sokoru district, respectively. The study revealed that the highest (42.9%) and lowest (7.5%) sero‐prevalence of the disease was recorded in Ambabesa Sadeka PA of Limmu Seka district and Haro Kake PA of Sokoru district, respectively. However, there was no statistically significant association (*p* < 0.05) of FMD sero‐prevalence among the nine PAs of the three districts, except in only Walake Sombo and Ambabesa Sadeka PAs of Limmu Kosa and Limmu Seka districts, respectively.

Out of the three randomly selected PAs of Limmu Kosa district, various sero‐prevalences were recorded with 40.9%, 22.5% and 28.6% in Walake Sombo, Debelo, Gena Dembi, respectively. Results of the analysis revealed that the highest PA level sero‐prevalence was observed in the Walake Sombo (40.9%) PA of the district. Therefore, the disease was statistically significant (*p* < 0.05) among animals examined from the Walake Sombo PA of Limmu Kosa districts in which the animals were more likely to be affected by FMD with more than four times (OR = 4.15; 95% CI: 1.5–11.9; *p* = 0.008) as compared with those animals examined from other PAs of the district (Table 3).

**TABLE 3:** Sero‐prevalence of FMD at the Level of PAs.

Selected districts	Peasant associations	No. of examined	No. of positive	Prevalence (%)	*P* value	SE	B	OR	95% CI	
									Lower	Upper
Limmu Kosa	Walake Sombo	44	18	40.91	0.008*	0.54	1.4	4.15	1.5	11.9
	Debelo	40	9	22.5	0.340	0.58	0.6	1.74	0.6	5.4
	Gena Dembi	42	12	28.57	0.117	0.55	0.9	2.40	0.8	7.2
Limmu Seka	A/Sadeka	49	21	42.86	0.004*	0.52	1.5	4.50	1.6	12.6
	Dame	43	10	23.25	0.294	0.57	0.6	1.82	0.6	5.6
	Dora	47	14	29.79	0.086	0.54	0.9	2.55	0.9	7.4
Sokoru	Tiro Shashama	37	4	10.81	0.644	0.68	−0.3	0.73	0.2	2.8
	Haro Kake	40	3	7.5	0.333	0.75	−0.7	0.49	0.1	2.1
	Adami Badeyi	42	6	14.29	—			—	—	—
Total		384	97	25.3	—			—	—	—

Note: PAs = * shows significance.

Like that of the Limmu Kosa district, three randomly selected PAs, namely, Ambabesa Sadeka, Dame and Dora of Limmu Seka districts, were included in the study to determine the sero‐prevalence of FMD at the PA level. As the result of the analysis indicated, different sero‐prevalences were recorded at 42.9%, 23.3% and 29.8% in Ambabesa Sadeka, Dame and Dora, respectively. That means the maximum sero‐prevalence of the disease was seen in Ambabesa Sadeka (42.9%). Therefore, the result showed that there is an association between the disease and PAs in which the animals were more likely to be affected by FMD more than four times (OR = 4.50; 95% CI: 1.6–12.6; *p* = 0.004).

On the other hand, a study was conducted in three randomly selected PAs, namely, Tiro Shashema, Haro Kake and Adami Badeyi of Sokoru district, and out of the examined animals of the PAs, the highest prevalence rate was recorded in the Adami Badeyi PA (14.3%). However, there was no statistically significant association (*p* < 0.05) of FMD sero‐prevalence among the three PAs of the district (Table 3).

### 3.3 Sero‐Prevalence of FMD With Respect to Age, Sex, Body Condition, Herd Size and Origin

A correlation between the disease and the sexes of the animals (male and female) was investigated; of the animals analysed, 208 (70%) were female and 176 (27%) were male. Females had a higher sero‐prevalence rate (33.7%) than males (15.3%). According to these data, female animals had an almost three‐fold higher likelihood of contracting that disease than did male animals (OR = 2.80; 95% CI: 1.7–4.6; *p* = 0.000). Sex was often shown to be significantly correlated (*p* < 0.05) with the prevalence of major cattle illnesses in the locations.

Conversely, an examination of the age‐specific sero‐prevalence of FMD in various age groups revealed nearly significant variations in prevalence rates within these age categories, with old, adult and young age groups exhibiting 39.1%, 24.0% and 8.0% of the total prevalence, respectively. According to this result, older age groups had higher disease prevalence than adult and younger age groups. In places where older age groups were more likely to be affected by the diseases more than six times as compared to adult and younger age groups, age generally exhibited a significant association with sero‐prevalence of FMD (OR = 6.3; 95% CI: 2.9–13.6; *p* = 0.000). The study animals were divided into three categories based on their physical state: good, medium and poor, with respective sero‐prevalences of 6.7%, 22.5% and 43.0%, respectively. The majority, however, was observed in animals with poor body condition, followed by those with medium body condition. The findings of the analysis indicated a statistically significant correlation (*p* < 0.05) between the disease and the physical state of the animals in the studied areas.

In this study, the animals' herd sizes were also examined; they were classified as belonging to large, medium or small herds, with sero‐prevalences of 43.1%, 27.7%, and 5.4%, respectively. However, animals from big herd sizes had the highest sero‐prevalence, followed by those from medium herd sizes. However, animals' herd sizes demonstrated a statistically significant correlation with the sero‐prevalence of FMD in the areas where animals from large herd sizes had a greater likelihood of contracting the disease than animals from medium and small herd sizes (OR = 13.4; 95% CI: 5.4–33.3; *p* = 0.000). The study animals were classified as either purchased or born, with sero‐prevalences of 56.4% and 9.4%, respectively, based on their place of origin. The animals purchased from other areas had the highest sero‐prevalence compared to those that were born. According to the analysis, there was a statistically significant correlation between the disease and the animals' place of origin in the study areas. Purchased animals had an almost nine‐fold higher risk of contracting the disease than animals born in the study areas (OR = 8.8; 95% CI: 5.2–14.8; *p* = 0.000) (Table 4).

**TABLE 4:** Summary of FMD sero‐prevalence by different variables.

Variables	Category level	No. of examined	No. of positive	Prevalence (%)	*P* value	OR	SE	B	95% CI	
									Lower	Upper
Districts	Limmu Kosa	126	39	40.0	0.001	3.7	0.4	1.3	1.8	7.3
	Limmu Seka	139	45	32.4	0.001	3.9	0.3	1.4	1.9	7.7
	Sokoru	119	13	10.9	—	—			—	—
Sex	Female	208	70	33.7	0.001	2.8	0.3	1.02	1.7	4.6
	Male	176	27	15.3	—	—			—	—
Age	Old	138	54	39.1	0.001	6.3	0.4	2	2.9	13.6
	Adult	146	35	24.0	0.004	3.2	0.3	0.7	1.5	6.7
	Young	100	8	8	—	—			—	—
Body condition	Poor	116	51	44.0	0.001	10.9	0.5	2.4	4.4	27.2
	Medium	178	40	22.5	0.002	4.1	0.3	0.9	1.6	9.9
	Good	90	6	6.7	—	—			—	—
Herd size of animal	Large	102	44	43.1	0.001	13.4	0.5	2.4	5.4	33.3
	Medium	170	47	27.7	0.009	6.8	0.3	0.7	2.8	16.4
	Small	112	6	5.4	—	—			—	—
Origin	Purchased	110	62	56.4	0.001	8.8	0.6	2.2`	5.2	14.8
	Born	274	35	9.4	—	—			—	—
Total		384	97	25.3	—	—			—	—

### 3.4 Herd Level Sero‐Prevalence of FMD

The districts also looked into FMD sero‐prevalence at the herd level. Thirty‐two of the 72 herds that were analysed tested positive for FMDv, and the study districts had an overall sero‐prevalence of 44.4% for the virus. In Limmu Kosa, Limmu Seka and Sokoru districts, the herd level sero‐prevalence was 52% (*n* = 25), 55.6% (*n* = 27) and 20% (*n* = 20) at the district level, respectively. The findings showed that Limmu Seka district had the comparatively greatest sero‐prevalence (55.6%), while Sokoru district had the lowest (20%). As a result, the herd‐level study revealed a statistically significant association (*p* < 0.05) between the sero‐prevalence of FMD in the districts where the disease was more likely to affect the herds examined from Limmu Seka district five times (OR = 5.0; 95% CI: 1.32–18.96; *p* = 0.018) and Limmu Kosa district more than four times (OR = 4.3; 95% CI: 1.13–16.68; *p* = 0.033) (Table 5).

**TABLE 5:** Sero‐prevalence of FMD by herd size of cattle and study districts.

Variable	Category level	No. of examined	No. of positive	Prevalence (%)	*P* value	OR	95% CI	
							Lower	Upper
District	Limmu Kosa	25	13	52	0.033	4.33	1.1	16.7
	Limmu Seka	27	15	55.6	0.018	5.0	1.3	18.9
	Sokoru	20	4	20	—	—	—	—
Herd size	Large	22	14	63.6	0.021	5.3	1.5	18.7
	Medium	26	12	46.2	0.307	2.6	0.8	8.6
	Small	24	6	25	—	—	—	—
Total		72	32	44.4				

In this study, the animals were divided into three herd sizes depending on the size of their herds: large, medium and small herds, with sero‐prevalences of 63.6% (14/22), 46.2% (12/26) and 25% (6/24), respectively. The results showed that the largest herd size, followed by the medium herd size, had the highest sero‐prevalence. Nonetheless, herd size had a statistically significant correlation with the sero‐prevalence of FMD in the regions where big herd sizes were five times more probable than medium and small herd sizes to be impacted by the illness (OR = 5.0; 95% CI: 1.5–18.7; *p* = 0.021). While the study classified the animals into large, medium and small herds in each district, the sero‐prevalence of FMD was found to be 83.3%, 55.6%, and 30% in Limmu Kosa; 63.6%, 55.6%, and 42.9% in Limmu Seka; and 40%, 25%, and 0% in Sokoru district, respectively. The results showed that the largest sero‐prevalence was observed in big herd sizes in Limmu Kosa, Limmu Seka and Sokoru districts, followed by medium herd sizes.

The disease herd‐level sero‐prevalence at the PA level was seen in Walke Sombo, Debelo, Gena Dembi, Ambabesa Sadeka, Dame, Dora, Tiro Shashema, Haro Kake and Adami Badeyi, with respective rates of 54.5%, 33.3%, 80%, 50%, 42.9%, 75%, 12.5%, 20% and 28.6%. The results indicated that the sero‐prevalence of the herd level was highest (80%) in Gena Dembi and lowest (12.5%) in Tiro Shashema PAs. However, among the PAs included in the analysis, there was no statistically significant correlation (*p* > 0.05) in the herd‐level sero‐prevalence of the disease. Additionally, during this study, the animals' herd sizes which were classified as large, medium and small herds in each PA were used to determine the sero‐prevalence of FMD. The results showed that the sero‐prevalence of the disease was, in Walake Sombo, 100%, 66.7% and 0% in Debelo, 100%, 100% and 0% in Gena Dembi, 25%, 66.7% and 60% in Ambabesa Sadeka, 50%, 50% and 0% in Dame, 100%, 50% and 0% in Dora, 0%, 25% and 0% in Tiro Shashema, 0%, 33.3% and 0% in Haro Kake, and 66.7%, 0% and 0% in Adami Badeyi PA, respectively. The results indicated that the herd size sero‐prevalence was highest (100%) in the Tiro Shashema PA and lowest (25%) in the Debelo, Gena Dembi, Dora and Ambabesa Sadeka PAs (Table 6).

**TABLE 6:** Sero‐prevalence of FMD by herd size of cattle in districts and PAs.

		Herd size									Total		
		Large			Medium			Small					
		Number of examined	Number of Positive	Prevalence (%)	Number of examined	Number of positive	Prevalence (%)	Number of examined	Number of positive	Prevalence (%)	Number of examined	Number of positive	Prevalence (%)
District	Limmu Kosa	6	5	5/6 (83.3)	9	5	5/9 (55.6)	10	3	3/10 (30)	25	13	13/25 (52)
	Limmu Seka	11	7	7/11 (63.6)	9	5	5/9 (55.6)	7	3	3/7 (42.9)	27	15	15/27 (55.6)
	Sokoru	5	2	2/5 (40)	8	2	2/8 (25)	7	0	0/7 (0)	20	4	4/20 (20)
PA's	Walake Sombo	2	1	½ (50)	3	2	2/3 (66.7)	6	3	3/6 (50)	11	6	6/11 (54.5)
	Debelo	1	1	1/1 (100)	5	2	2/5 (66.7)	3	0	0/3 (0)	9	3	3/9 (33.3)
	Gena Dembi	3	3	3/3 (100)	1	1	1/1(100)	1	0	0/1 (0)	5	4	4/5 (80)
	Ambebesa Sadeka	4	1	¼ (25)	3	2	2/3 (66.7)	5	3	3/5 (60)	12	6	6/12 (50)
	Dame	2	1	½ (50)	4	2	2/4 (50)	1	0	0/1 (0)	7	3	3/7 (42.9)
	Dora	5	5	5/5 (100)	2	1	½ (50)	1	0	0/1 (0)	8	6	6/8 (75)
	Tiro Shashema	1	0	0/1 (0)	4	1	¼ (25)	3	0	0/3 (0)	8	1	1/8 (12.5)
	Haro Kake	1	0	0/1 (0)	3	1	1/3 (33.3)	1	0	0/1 (0)	5	1	1/5 (20)
	Adami Badeyi	3	2	2/3 (66.7)	1	0	0/1 (0)	3	0	0/3 (0)	7	2	2/7 (28.6)
	Total	22	14	14/22 (63.3)	26	12	12/26 (46.2)	24	6	6/24 (25)	72	32	32/72 (44.4)

## 4 Discussions

In the current study, fieldwork and serological analysis were used to examine the exposure to the FMDv in the three administrative districts of the Jimma zone (Limmu Kosa, Limmu Seka and Sokoru) between December 2021 and July 2022. Age, sex, body condition, herd size and origin were among the risk factors taken into account in the current study district that were determined to be statistically significant (*p* < 0.05) in logistic regression.

Three districts of the Jimma zone were chosen at random, and results showed an overall sero‐prevalence of 25.3% at the animal level and 44.4% at the herd level. Due to the animals' lack of vaccination against the illness, this study showed that FMD is one of the most prevalent cattle diseases in the studied locations. Cattle's serum contained antibodies against the FMDV NSP, indicating that they had previously been exposed to a natural infection (OIE 2009). According to two recent studies, the prevalences of 25.3% and 24.2%, respectively, in Eastern Iraq and Ethiopia, were quite similar, as revealed by researchers (Sulayeman et al. 2018, Al‐Ajeeli et al. 2018). Comparable investigations carried out in various locations and at various times revealed varying prevalence. This finding's result was comparatively similar to previously reported prevalences of 21.4% and 26.5%, respectively, as reported by Rufael et al. (2008), Mekonen et al. (2011) and Fanta et al. (2014).

Furthermore, the current study's results at the individual animal level were in line with earlier findings regarding sero‐prevalence, which showed that 21% of the population in the Guji and Borana zones (OIE 2013), 21.4% in the Kellem Wollega zone (Fanta et al. 2014), 24.6% in Southern Ethiopia (Mekonen et al. 2011), 19.8% in Afar (Dubie and Negash 2021), 20.9% in Tigray (Taye, Afera, and Abebe 2020), 23% in the Borena pastoral and agro‐pastoral area (Bayissa et al. 2011), in the West Shewa zone, North Shewa zone and Addis Ababa with 30.8% (Beksisa 2017), 32.7% in the Guji zone of the Oromia region, 30% in the Yeka district city of Addis Ababa, and 28.3% sero‐positivity in the Akaki‐kality sub‐city (Dubie and Negash 2021, Taye, Afera, and Abebe 2020, Bayissa et al. 2011, Beksisa 2017, Negusssie et al. 2011).

Conversely, the sero‐prevalence recorded in this survey was higher than that of the earlier publications—Gelana and Abera (2016), Molla et al. (2010), Megersa et al. (2009), Ayelet et al. (2012), Gelaye et al. (2009), Yahya et al. (2013) and Abdulahi, Esaya, and Hailu (2011)—that reported 4.8%, 8.18%, 9.5%, 10.5%, 12.1%, 11.6% and 14.1%, respectively. Contrary to the present findings, earlier reports of relatively lower sero‐prevalence of FMD have been made with varying prevalence magnitudes, such as 13% in some Western Ethiopian districts (Asresie, Zemedu, and Adigrat 2015), 10.88% in some Eastern Showa zone, Oromia region districts, 5.53% on quarantined bulls for export at Adama and Dire Dawa stations, and 4.8% in some Western Oromia region districts, 5.6% in Afar, 8.01% in Dire Dawa, 11.48% in Amhara, and 10.88% in Bishoftu (Bedru 2006, Milkessa et al. 2016, Jenbere et al. 2011, Abunna, Fikru, and Rufael 2013, Mesfine et al. 2019, Belina, Girma, and Mengistu 2016). Geographic variation, the timing of an infection, the production system, the mobility of the herd in search of pasture and water, animal mixing at watering spots, the size of the herd and regular interactions with other animals and wildlife could all be contributing factors to this.

Conversely, greater sero‐prevalence values were reported from earlier investigations in the Eastern zone of Tigray (41.5%) and central Ethiopia (72.1%), Welmera (49.2%), West Shewa zone (40.4%), Oromia and Addis Ababa (38.6%), Borena (53.6%) and Borena (49.6%) (Awel et al. 2021, Beksisa et al. 2020, Ahmed et al. 2020, Urge 2017, OIE 2012). A study from neighbouring Sudan revealed that 79% of cattle had FMD sero‐prevalence (OIE 2012). Additionally Hafez et al. (1994) report that FMD sero‐positivity was observed in Saudi Arabia with a 53% sero‐prevalence, while Namatovu et al. (2015) report that 77% sero‐prevalence was observed in Uganda from infected cattle. These differences from the current findings regarding sero‐positivity variation in FMD sero‐prevalence results could be attributed to various factors, including the type of diagnostic tests used, the sampling method, study areas, geographic variation and timing of infection, and the production system, which is characterized by high herd mobility in search of pasture and water, animal intermingling at watering points, large herd sizes, and frequent cross‐border contact with livestock from neighbouring countries as well as regular livestock contact with wildlife, including buffalo, wild pigs, kudu and warthog, among other factors (Gelaye et al. 2009, Megersa et al. 2009).

The findings showed that the sero‐prevalence of the illness in cattle in the districts of Limmu Kosa, Limmu Seka and Sokoru was 30.9%, 32.4%, and 10.3%, respectively. Thus, compared to animals sampled from Sokoru district, the disease was statistically significant (*p* < 0.05) among animals from Limmu Seka, where the animals were nearly four times more likely to be affected by FMD (OR = 3.9; 95% CI: 1.9–7.7; *p* = 0.000) and Limmu Kosa districts (OR = 3.70; 95% CI: 1.8–7.3; *p* = 0.000). In general, there was a strong correlation (*p* < 0.05) between the district and the prevalence of important livestock diseases in the areas. The highest sero‐prevalence was found in the districts of Limmu Kosa and Limmu Seka, which is likely due to the higher pattern of contact between domestic animals and wild animals. Additionally, the Jimma zone borders the East Wollega, West Shoa, Illuababor zone and SNNP region, where it is possible that cattle movement across borders occurs illegally, supporting the national theory that the primary cause of FMD transmission is cattle movement, which results in contact with cattle from different origins, as reported (Rufael et al. 2008).

The results align with other studies (Milkessa et al. 2016), which highlight the notable differences in the Gobu‐Sayo (0.8%) and Horro (8.2%) districts of the western Oromia regional state. This could be because each administrative organization has a different style of grazing, and there are variations in the movement and distribution of livestock as well as the degree of interaction between herds and ungulate fauna. Unrestricted animal movement in search of grazing and drinking spots; throughout the majority of the nation, animals are frequently carried on foot to local market destinations, which contributes to the spread of the infection. Previous research (Abunna, Fikru, and Rufael 2013, Mersie et al. 1992) showed that the rate of infection is increased by the large amount of cattle movement and the high rate of animal‐to‐animal contact during marketing, common grazing areas and watering spots.

Females had a greater sero‐prevalence of FMDV (32.5%) compared to males (16.98%). According to these data, female animals had an almost three‐fold higher likelihood of being impacted by FMDV than male cattle (OR = 2.8; 95% CI: 1.697–4.619, P = 0.001). In general, there was a strong correlation (*p* < 0.05) between sex and the prevalence of important cattle illnesses in the area. The results of the study indicated a relationship between sex and FMD prevalence in cattle, with female cattle having the highest incidence of the disease compared to male cattle. The results were consistent with the data of (Mazengia et al. 2010), which states that FMD is more common in female cattle (16.63%) than in male cattle (1.37%). Moreover, comparable observations (Mekonen et al. 2011, Mesfine et al. 2019, Mazengia et al. 2010) revealed that females had a greater sero‐prevalence of bovine FMD than male. Nonetheless, the results diverged from those of other investigators, who reported a greater incidence in male animals as opposed to female animals (Remond, Kaiser, and Leberton 2002, Sarker et al. 2011).

According to Chepkwony et al. (2012) and Gelaye et al. (2009), this result was lower than prior research in Kenya, where there were 67% in female cattle and 33% in male cattle. It was also greater than previous discoveries in Ethiopia, where there were 8.27% in male and 15.07% in female cattle. Given that females experience more physiological stress than males do, it is possible that this accounts for the notable variance in sero‐prevalence between the sexes of cattle. Due to physiological stressors including oestrus, pregnancy and lactation, which are known to impair an individual's immunity to infection, females may have a higher percentage of sero‐positivity (Susan and Asamays 1998). Similarly, the Benjie Maji regions of southern Ethiopia were found to have a higher FMD sero‐prevalence in females (Gelaye et al. 2009). For breeding purposes, females are maintained longer than males, which could lead to a higher sero‐prevalence of FMD in females.

There was practically a difference in the sero‐prevalence rate between these age categories, which was 39.1%, 24%, and 8% in the old, adult and young, respectively, according to an analysis of the age‐wise sero‐prevalence of FMD in cattle. According to this result, older age groups had a higher disease prevalence than adult and younger age groups. In places where older age groups were more likely to be affected by the diseases more than six times as compared to adult and younger age groups, age generally exhibited a significant association with sero‐prevalence of FMD (OR = 6.3; 95% CI: 2.93–13.56; *p* = 0.000). Previous publications (Rufael et al. 2008, Megersa et al. 2009, Molla et al. 2010, Negusssie et al. 2011, Yahya et al. 2013, Kibore et al. 2013) that suggested sero‐positivity increases with age corroborate this finding. The current study's significant sero‐prevalence in older adults may be due to a history of illness without vaccination or immunization, as well as a failure to confine diseased adult and older animals separately. Adults and older animals may also be driven freely into grazing and drinking areas where interaction with other affected animals can spread the infection. These circumstances facilitate the disease's development and subsequent spread among mature, aged cattle.

The young's low sero‐prevalence could be attributed to passive maternal immunity, which shields them from the illness. So as to decrease the risk of infection. The results of this study, which showed a statistically significant higher sero‐prevalence of FMDV in old and adult animals than in young cattle, were in close agreement with findings from earlier studies by Asresie, Zemedu, and Adigrat (2015), who found that adult cattle in western Ethiopia were 2.7 times more likely than young cattle to contract the disease in Borna pastoral and agro‐pastoral area, South Omo zone, Gamogofa and Sidama zones, Awbere and Babille districts of Jijiga zone, and South Omo zone. Additionally, there was a strong agreement between this age connection and FMD serostatus and a prior study by Ocaido et al. (2009). Age may be related to FMD sero‐prevalence for a few reasons. First, older and adult cattle may have contracted the illness from repeated exposure to different virus serotypes over time, and they may have mixed with other herds at public pastures and markets. Moreover, it could be caused by antibodies that have a long half‐life against FMD NSP (Tesfaye et al. 2016).

Relatively reduced sero‐prevalence in animal groups less than three years old may indicate low exposure frequency and the presence of passive maternal immunity (Abdulahi, Esaya, and Hailu 2011, Jenbere et al. 2011). Because of the prevalent passive maternal immunity, young cattle in the current study locations are less likely to be exposed to the virus and may therefore be protected from the illness. Conversely, the study's findings ran counter to those of Gelaye et al. (2009) and Esayas et al. (2009), who found no evidence of a significant correlation between cattle age in the Bench Maji zone of southern Ethiopia and FMD sero‐positivity. According to 74 as age increases, the chance of exposure to the disease increases. This might be because olds and adults have acquired the infection through repeated exposure to the different serotypes of the virus and could get access to mix with other herds at market places and communal pasture land. Aged animals might have acquired the infection from multiple serotypes, and could have produced antibodies against those serotypes. Relatively low sero‐prevalence in animal groups below two years old might be indicative of the existence of passive maternal immunity and low frequency of exposure (Negusssie et al. 2011, Gelana and Abera 2016, Molla et al. 2010, Yahya et al. 2013, Abdulahi, Esaya, and Hailu 2011, Belina, Girma, and Mengistu 2016, Bedru 2006, Milkessa et al. 2016, Jenbere et al. 2011, Abunna, Fikru, and Rufael 2013, Mesfine et al. 2019, Belina, Girma, and Mengistu 2016, Awel et al. 2021, Beksisa et al. 2020, Ahmed et al. 2020, Urge 2017, OIE 2012, Hafez et al. 1994, Namatovu et al. 2015, Mersie et al. 1992, Mazengia et al. 2010, Remond, Kaiser, and Leberton 2002, Sarker et al. 2011, Chepkwony et al. 2012, Susan and Asamays 1998, Kibore et al. 2013, Ocaido et al. 2009, Tesfaye et al. 2016, Esayas et al. 2009, Murphy et al. 1999, Bayissa 2009).

Cattle were divided into three categories based on their BCS: poor, medium and good. The highest sero‐prevalence (44%) was seen in animals with poor body condition ratings, compared to medium (22.5%) and good (6.7%) scores. However, the animals with the poorest body condition showed the highest prevalence, followed by those with the medium body condition. The results of the analysis showed that there was a substantial (*p* < 0.05) correlation between the animals' physical conditions and the disease in the study locations. The report of Fraser (1991) provides additional support for the outcome of this finding. Given that animals in good physical condition have a reasonably good immune response to infection, it is likely that the inadequate protective immune response in poorly conditioned cattle is the cause (Radostits et al. 2007). Animals with medium and poor body conditions showed a considerably greater prevalence (*p* = 0.000), respectively. This may be because of the disease's crippling effects on an animal's physical condition (Tesfaye 2006) or possibly because of other concurrent diseases that weaken an animal's immune and increase its susceptibility to FMD.

The study also looked into the size of the animal herds, classifying the animals into large, medium and small herds based on their respective sero‐prevalences of 43.1%, 27.7%, and 5.4%. However, animals from big herd sizes exhibited the highest sero‐prevalence, followed by those from medium herd sizes. However, the herd size of the animals exhibited a statistically significant correlation with the sero‐prevalence of FMD in the areas where the animals from large herd sizes had a greater likelihood of contracting the disease than the animals from medium and small herd sizes (OR = 13.4; 95% CI: 5.4–33.3; *p* = 0.000). This indicates that when herd size increased, so did the sero‐prevalence of antibodies against FMD NSP. According to this finding, animals from medium and large herd sizes had a considerably higher chance of contracting that disease (*p* = 0.000) than animals from small herd sizes.

According to the current study's findings, when all other characteristics were held constant, animals from big and medium herd sizes, respectively, were considerably more likely to get the disease than animals from small herd sizes (*p* < 0.05). The current results were consistent with those of Gelaye et al. (2009), Bayissa et al. (2011), Asresie, Zemedu, and Adigrat (2015), Dubie and Negash (2021) and Bayissa et al. (2011), who found a positive correlation between FMD sero‐prevalence and herd size. The infectious nature of the disease and its mode of transmission, which is linked to animal overpopulation, may be indicated by this direct association. This could increase the possibility of transmission by facilitating frequent direct contact. Nonetheless, the current investigation demonstrated the significance of herd size in the disease's epidemiology. Given that the disease is contagious, direct interactions and the route of transmission may be to blame for this (Seifert 1996). Excessive herd sizes may result in animal crowding, which increases the frequency of encounters and, ultimately, the risk of transmission.

The districts also looked into the FMD herd‐level sero‐prevalence. In the study districts, there was an overall sero‐prevalence of 44.4% for FMDV, out of the 72 herds studied, 32 of which tested positive for the virus. The district‐level herd‐level sero‐prevalence in Limmu Kosa, Limmu Seka and Sokoru districts was 52% (*n* = 25), 55.6% (*n* = 27), and 20% (*n* = 20), respectively. The findings showed that Limmu Seka district had the comparatively greatest sero‐prevalence (55.6%), while Sokoru district had the lowest (20%). As a result, the herd‐level study revealed a statistically significant association (*p* < 0.05) between the sero‐prevalence of FMD in the districts where the disease was more likely to affect the herds examined from Limmu Seka district five times (OR = 5.0; 95% CI: 1.3–18.9; *p* = 0.018) and Limmu Kosa district more than four times (OR = 4.3; 95% CI: 1.1–16.7; *p* = 0.033). The results were consistent with other sero‐prevalence studies by Dubie and Negash (2021), Bayissa et al. (2011) and Tesfaye (2006), which found sero‐prevalence rates of 59%, 58.6% and 59.6%, respectively, in Borna pastoral and agro‐pastoral areas and districts of the Afar region. This might be due to the fact that the occurrence of an outbreak last year in Limmu Kosa, the presence of a high reaction of livestock market in the area, high herd contact in grazing and watering area and a high population of small ruminants moving from place to place. This suggests that small ruminants and herd contacts may have an important role in the epidemiology of FMD as they can serve as potential carriers and transmitters of the disease (Abdulahi, Esaya, and Hailu 2011, Jenbere et al. 2011).

In this study, the animals' herd sizes were used to determine the sero‐prevalence of FMD. The animals were classified as large, medium and small herds, with sero‐prevalences of 63.6% (14/22), 46.2% (12/26) and 25% (6/24), respectively. The results showed that big herd sizes had the highest sero‐prevalence, followed by medium herd sizes. Herd size did, however, demonstrate a statistically significant correlation with the sero‐prevalence of FMD in the locations where it was found that big herd sizes were five times more likely to be afflicted with the disease than medium and small herd sizes (OR = 5.0; 95% CI: 1.5–18.7; P = 0.021). The current results corroborated those of (Gelaye et al. 2009, Bayissa et al. 2011, Bayissa et al. 2011), who found a positive correlation between FMD sero‐prevalence and herd size. The infectious nature of the disease and its mode of transmission, which is linked to animal overpopulation, may be indicated by this direct association. This could increase the possibility of transmission by facilitating frequent direct contact. Nonetheless, the current investigation demonstrated the significance of herd size in the disease's epidemiology. Given that the disease is contagious, direct interactions and the route of transmission (Seifert 1996) may be to blame for this. Excessive herd sizes may result in animal crowding, which increases the frequency of encounters and, ultimately, the risk of transmission (supporting information).

An effort was made to observe herd‐level sero‐prevalence of the disease at the PA level with 54.5%, 33.3%, 80%, 50%, 42.9%, 75%, 12.5%, 20% and 28.6% in Walke Sombo, Debelo, Gena Dembi, Ambabesa Sadeka, Dame, Dora, Tiro Shashema, Haro Kake and Adami Badeyi, respectively. The finding showed that the highest (80%) and lowest (12.5%) herd‐level sero‐prevalence was observed in Gena Dembi and Tiro Shashema PAs, respectively. However, there was no statistically significant association (*p* > 0.05) in herd‐level sero‐prevalence of the disease among the PAs included in the study. The presence of sero‐positive herds in all PAs could also be in support of herd‐level endemicity of FMD in the Borana area (Rufael et al. 2008). Moreover, the significant difference between overall sero‐prevalence of 80%, 54.5% and 33.3% in three PAs of Limmu Kosa district; 75%, 50% and 42.9% in three PAs of Limmu Seka district and 28.6%, 20% and 12.5% in the other three PAs in Sokoru District recorded might be associated with frequent contacts of animals during high livestock trade activities, thus having livestock frequently mix with animals coming from different area.

Depending on their origin, study animals were grouped into two as the home born and purchased with sero‐prevalence of 9.4% and 56.4%, respectively. However, the highest sero‐prevalence was seen in animals purchased from other areas than animals home born. This result indicated that purchased animals were more likely to be affected by those diseases almost nine times as compared to that of home‐born animals (OR = 8.8; 95% CI: 5.2–14.8; *p* = 0.000). Generally, origin showed a significant association between the disease and origin of the animals in the study areas (*p* < 0.05). Previous studies (Abunna, Fikru, and Rufael 2013, Mersie et al. 1992) indicated that extensive movement of livestock and the high rate of contact between animals at marketing and common grazing places as well as at watering points increase the rate of infection. In the current study, the higher sero‐prevalence in purchased cattle than home born cattle could be due to contacts of hundreds of cattle at local open‐air markets from different farmers, villages, towns, PAs, districts, and zones especially during the ceremony which were then taken back to different localities that certainly facilitate the spread of FMDv among cattle in the area. The present finding showed the purchase of live cattle from an open market is the main risk factor for FMDV dissemination.

FMD is one of the most common diseases that can cause restrictions on the trade of animals both locally and internationally, thereby threatening the livelihood of farmers in particular and the national agricultural economy in general (Rufael 2006). The increase in prevalence may be associated with extensive movement of livestock and the high rate of contact between animals at marketing and common grazing places as well as at watering points (Mersie et al. 1992). The increase of prevalence in purchased cattle may be due to the fact that, in some areas, cattle have to move long distances in search of higher payment offered by middlemen. This movement causes stress in the animals and leads to contact between cattle of different origins, which is the predominant factor for the transmission of the disease. Additionally, in the study districts, the livestock production system was extensive system; animals used communal grazing land and watering points. The free animal movement was a common practice in the area. The introduction of new animals into individually owned herds following purchase for replacement, herd expansion and receiving cultural gifts was usually exercised without any screening for the health status of the new animal.

## 5 Conclusion and Recommendations

FMD is endemic across several zones in most regions of Ethiopia with different prevalences. The findings of the present study revealed that FMD are highly prevalent in selected districts of Jimma zone. Even with the absence of an outbreak, 25.3% sero‐prevalence of the disease was recorded. It indicates that the cattle population of the study area harbours carrier individuals that could lead the virus to strike again in the population. Farmers appear to have good knowledge of the manifestation, seasonality and economic impact of FMD but are not aware of the interspecies transmission of the disease between shoats, wildlife and due to contacts at watering and grazing points. Generally, the current study ascertained and explained the continuity of FMD introduction and the endemic situation of the disease with widely distributed and identified associated risk factors in the study areas. Therefore, about the concluding remarks, recommendations are pointed out: Identification of circulating serotypes (strains) of FMD in the areas should be further studied to undertake vaccination programme; farmers and livestock owners should be trained about the disease, husbandry practices and to report the FMD outbreak; successful control and prevention strategies such as annual vaccination and biosecurity; and eradication preparations should be implemented throughout the country; effective surveillance systems for timely information about the occurrence, spread and causative serotypes are mandatory for efficient control and separation of animals at grazing and watering points should be promoted to reduce the spread of disease from one animal to another animal.

## Author Contributions

**Geremew Esema**: Have made significant contributions to conception and design, acquisition of data, analysis and interpretation of data and writing‐original draft; **Zelalem Abera**: Have made important contributions to conception and design, supervision, analysis and interpretation of data and revising the manuscript critically for important intellectual content. **Moti Wakgari**: Been involved in conception and design, acquisition of data, Supervision and revising the manuscript. **Eshetu Gezahegn**: Been involved in conception and design, acquisition of data, Supervision and revising the manuscript.

## Ethics Approval and Consent to Participate

Ethical approval (A formal letter) for this study was granted from Wollega University; Department of Veterinary Clinical Science (VCSc) and Laboratory Technology. Before starting data collection, oral consent was obtained from the animal's owners after verbal informed consent was approved by the Wallaga University ethics committee. The participants were informed about the purpose of the study.

## Conflicts of Interest

The authors declare no conflicts of interest.

### Peer Review

The peer review history for this article is available at https://publons.com/publon/10.1002/vms3.70239

## Supporting information



Supporting Information

Supporting Information

## Data Availability

The data collected and used to support this article can be offered by the first or corresponding author upon request.
