# Supplementary material for: Sero‐Prevalence of Foot‐and‐Mouth Disease in Cattle in Selected Districts of Jimma Zone, South‐Western Ethiopia
Source: Vet Med Sci. 2025 Feb 22;11(2):e70239. doi: 10.1002/vms3.70239 (PMC11846150; doi:10.1002/vms3.70239)
Supplement: Supplementary file 1 — Supporting Information [file VMS3-11-e70239-s002.docx]

**Supplementary Figures**


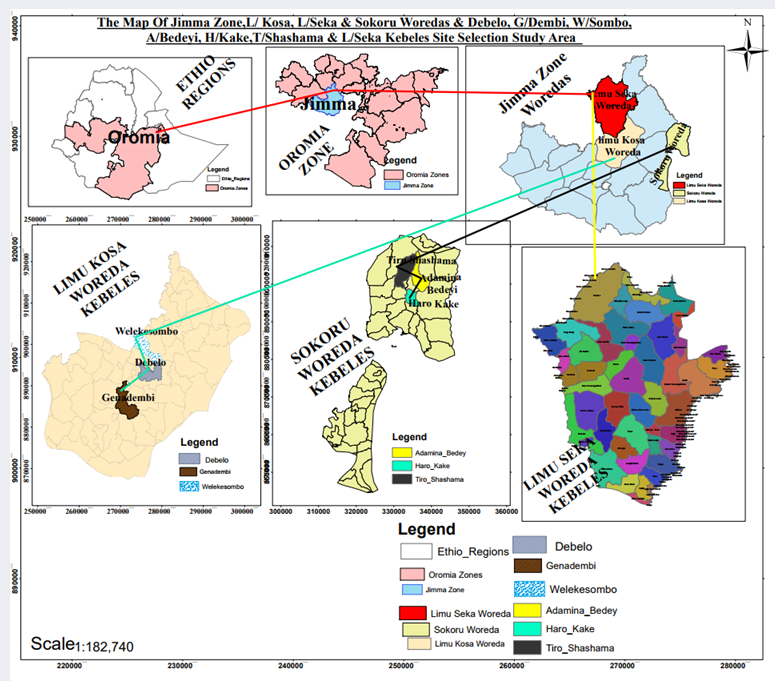


Supplementary Figure 1. Map of study area (Projected using ArcGIS software)
