# Supplementary material for: Sero‐Prevalence of Foot‐and‐Mouth Disease in Cattle in Selected Districts of Jimma Zone, South‐Western Ethiopia
Source: Vet Med Sci. 2025 Feb 22;11(2):e70239. doi: 10.1002/vms3.70239 (PMC11846150; doi:10.1002/vms3.70239)
Supplement: Supplementary file 2 — Supporting Information [file VMS3-11-e70239-s001.docx]

**Supplementary Tables**

**Supplementary Table 1.** Population of cattle and number of households per PA in the selected PA

| District | Peasant association (PA) | Number of households/PA | Number of cattle/PA | Number of selected Households | Number of samples taken per PA |
| --- | --- | --- | --- | --- | --- |
| Limmu Kosa | Waleke Sombo | 652 | 5454 | 13 | 44 |
|  | Debelo | 594 | 4328 | 9 | 40 |
|  | Gena Dembi | 708 | 3668 | 7 | 42 |
| Limmu  Seka | Ambabesa Sadeka | 680 | 6728 | 15 | 49 |
|  | Dora | 517 | 4809 | 11 | 43 |
|  | Dame | 449 | 3586 | 10 | 47 |
| Sokoru | Tiro Shashema | 565 | 4584 | 12 | 37 |
|  | Haro Kake | 532 | 3298 | 11 | 40 |
|  | Adami Badeyi | 687 | 4199 | 12 | 42 |
| Total | | | | 100 | 384 |

**Supplementary Table 2. Sero-prevalence of FMD at district level**

| **Study Districts** | **N^o^ of examined** | **N^o^ of Positive** | **Prevalence (%)** | **P-value** | **OR** | **95% CI** | |
| --- | --- | --- | --- | --- | --- | --- | --- |
|  |  |  |  |  |  | **Lower** | **Upper** |
| Limmu Kosa | 126 | 39 | 31 | 0.000 | 3.7 | 1.8 | 7.3 |
| Limmu Seka | 139 | 45 | 32.4 | 0.000 | 3.9 | 1.9 | 7.7 |
| Sokoru | 119 | 13 | 10.9 | - | - | - | - |
| **Total** | **384** | **97** | **25.3** | **-** | **-** | **-** | **-** |

**Supplementary Table 3**: Sero-prevalence of FMD at the Level of Peasant Associations (PAs)

| **Selected Districts** | **Peasant Associations** | **N^o^of examined** | **N^o^of Positive** | **Prevalence (%)** | **P-value** | **S.E** | **B** | **OR** | **95% CI** | |
| --- | --- | --- | --- | --- | --- | --- | --- | --- | --- | --- |
|  |  |  |  |  |  |  |  |  | **Lower** | **Upper** |
| Limmu Kosa | Walake Sombo | 44 | 18 | 40.91 | 0.008* | 0.54 | 1.4 | 4.15 | 1.5 | 11.9 |
|  | Debelo | 40 | 9 | 22.5 | 0.340 | 0.58 | 0.6 | 1.74 | 0.6 | 5.4 |
|  | Gena Dembi | 42 | 12 | 28.57 | 0.117 | 0.55 | 0.9 | 2.40 | 0.8 | 7.2 |
| Limmu Seka | A/Sadeka | 49 | 21 | 42.86 | 0.004* | 0.52 | 1.5 | 4.50 | 1.6 | 12.6 |
|  | Dame | 43 | 10 | 23.25 | 0.294 | 0.57 | 0.6 | 1.82 | 0.6 | 5.6 |
|  | Dora | 47 | 14 | 29.79 | 0.086 | 0.54 | 0.9 | 2.55 | 0.9 | 7.4 |
| Sokoru | Tiro Shashama | 37 | 4 | 10.81 | 0.644 | 0.68 | -0.3 | 0.73 | 0.2 | 2.8 |
|  | Haro Kake | 40 | 3 | 7.5 | 0.333 | 0.75 | -0.7 | 0.49 | 0.1 | 2.1 |
|  | Adami Badeyi | 42 | 6 | 14.29 | - |  |  | - | - | - |
| **Total** |  | **384** | **97** | **25.3** | **-** |  |  | **-** | **-** | **-** |

**NB:** PAs = *shows significance

Supplementary Table 4: Summary of FMD Sero-prevalence by different variables

| **Variables** | **Category**  **level** | **N^o^ of**  **examined** | **N^o^ of**  **Positive** | **Prevalence (%)** | **P-value** | **OR** | **S.E** | **B** | **95% CI** | |
| --- | --- | --- | --- | --- | --- | --- | --- | --- | --- | --- |
|  |  |  |  |  |  |  |  |  | **Lower** | **Upper** |
| Districts | Limmu Kosa | 126 | 39 | 40.0 | 0.001 | 3.7 | 0.4 | 1.3 | 1.8 | 7.3 |
|  | Limmu Seka | 139 | 45 | 32.4 | 0.001 | 3.9 | 0.3 | 1.4 | 1.9 | 7.7 |
|  | Sokoru | 119 | 13 | 10.9 | - | - |  |  | - | - |
| Sex | Female | 208 | 70 | 33.7 | 0.001 | 2.8 | 0.3 | 1.02 | 1.7 | 4.6 |
|  | Male | 176 | 27 | 15.3 | - | - |  |  | - | - |
| Age | Old | 138 | 54 | 39.1 | 0.001 | 6.3 | 0.4 | 2 | 2.9 | 13.6 |
|  | Adult | 146 | 35 | 24.0 | 0.004 | 3.2 | 0.3 | 0.7 | 1.5 | 6.7 |
|  | Young | 100 | 8 | 8 | - | - |  |  | - | - |
| Body condition | Poor | 116 | 51 | 44.0 | 0.001 | 10.9 | 0.5 | 2.4 | 4.4 | 27.2 |
|  | Medium | 178 | 40 | 22.5 | 0.002 | 4.1 | 0.3 | 0.9 | 1.6 | 9.9 |
|  | Good | 90 | 6 | 6.7 | - | - |  |  | - | - |
| Herd size of animal | Large | 102 | 44 | 43.1 | 0.001 | 13.4 | 0.5 | 2.4 | 5.4 | 33.3 |
|  | Medium | 170 | 47 | 27.7 | 0.009 | 6.8 | 0.3 | 0.7 | 2.8 | 16.4 |
|  | Small | 112 | 6 | 5.4 | - | - |  |  | - | - |
| Origin | Purchased | 110 | 62 | 56.4 | 0.001 | 8.8 | 0.6 | 2.2` | 5.2 | 14.8 |
|  | Born | 274 | 35 | 9.4 | - | - |  |  | - | - |
| **Total** | | **384** | **97** | **25.3** | **-** | **-** |  | | **-** | **-** |

Supplementary Table 5: Sero-prevalence of Foot and Mouth Disease by herd size of cattle and study districts

| **Variable** | **Category level** | **No. of examined** | **No. of positive** | **Prevalence (%)** | **P-value** | **OR** | **95% CI** | |
| --- | --- | --- | --- | --- | --- | --- | --- | --- |
|  |  |  |  |  |  |  | Lower | Upper |
| District | Limmu Kosa | 25 | 13 | 52 | 0.033 | 4.33 | 1.1 | 16.7 |
|  | Limmu Seka | 27 | 15 | 55.6 | 0.018 | 5.0 | 1.3 | 18.9 |
|  | Sokoru | 20 | 4 | 20 | - | - | - | - |
| Herd size | Large | 22 | 14 | 63.6 | 0.021 | 5.3 | 1.5 | 18.7 |
|  | Medium | 26 | 12 | 46.2 | 0.307 | 2.6 | 0.8 | 8.6 |
|  | Small | 24 | 6 | 25 | - | - | - | - |
| **Total** | | **72** | **32** | **44.4** |  |  |  |  |

Supplementary Table 6: Sero-prevalence of FMD by herd size of cattle in districts and PAs

|  | | **Herd size** | | | | | | | | | **Total** | | |
| --- | --- | --- | --- | --- | --- | --- | --- | --- | --- | --- | --- | --- | --- |
|  |  | **Large** | | | **Medium** | | | **Small** | | |  |  |  |
|  |  | Number of  examined | Number of  Positive | Prevalence (%) | Number of  examined | Number of  Positive | Prevalence (%) | Number of  examined | Number of  Positive | Prevalence (%) | Number of  examined | Number of  Positive | Prevalence (%) |
| District | Limmu Kosa | 6 | 5 | 5/6 (83.3) | 9 | 5 | 5/9 (55.6) | 10 | 3 | 3/10 (30) | 25 | 13 | 13/25 (52) |
|  | Limmu Seka | 11 | 7 | 7/11 (63.6) | 9 | 5 | 5/9 (55.6) | 7 | 3 | 3/7 (42.9) | 27 | 15 | 15/27 (55.6) |
|  | Sokoru | 5 | 2 | 2/5 (40) | 8 | 2 | 2/8 (25) | 7 | 0 | 0/7 (0) | 20 | 4 | 4/20 (20) |
| PA’s | Walake Sombo | 2 | 1 | ½ (50) | 3 | 2 | 2/3 (66.7) | 6 | 3 | 3/6 (50) | 11 | 6 | 6/11 (54.5) |
|  | Debelo | 1 | 1 | 1/1 (100) | 5 | 2 | 2/5 (66.7) | 3 | 0 | 0/3 (0) | 9 | 3 | 3/9 (33.3) |
|  | Gena Dembi | 3 | 3 | 3/3 (100) | 1 | 1 | 1/1(100) | 1 | 0 | 0/1 (0) | 5 | 4 | 4/5 (80) |
|  | Ambebesa Sadeka | 4 | 1 | ¼ (25) | 3 | 2 | 2/3 (66.7) | 5 | 3 | 3/5 (60) | 12 | 6 | 6/12 (50) |
|  | Dame | 2 | 1 | ½ (50) | 4 | 2 | 2/4 (50) | 1 | 0 | 0/1 (0) | 7 | 3 | 3/7 (42.9) |
|  | Dora | 5 | 5 | 5/5 (100) | 2 | 1 | ½ (50) | 1 | 0 | 0/1 (0) | 8 | 6 | 6/8 (75) |
|  | Tiro Shashema | 1 | 0 | 0/1 (0) | 4 | 1 | ¼ (25) | 3 | 0 | 0/3 (0) | 8 | 1 | 1/8 (12.5) |
|  | Haro Kake | 1 | 0 | 0/1 (0) | 3 | 1 | 1/3 (33.3) | 1 | 0 | 0/1 (0) | 5 | 1 | 1/5 (20) |
|  | Adami Badeyi | 3 | 2 | 2/3 (66.7) | 1 | 0 | 0/1 (0) | 3 | 0 | 0/3 (0) | 7 | 2 | 2/7 (28.6) |
|  | **Total** | **22** | **14** | **14/22 (63.3)** | **26** | **12** | **12/26 (46.2)** | **24** | **6** | **6/24 (25)** | **72** | **32** | **32/72 (44.4)** |
